# Modelling the lifetime cost-effectiveness of radical prostatectomy, radiotherapy and active monitoring for men with clinically localised prostate cancer from median 10-year outcomes in the ProtecT randomised trial

**DOI:** 10.1186/s12885-020-07276-4

**Published:** 2020-10-07

**Authors:** S. Sanghera, S. Mohiuddin, J. Coast, K. Garfield, S. Noble, C. Metcalfe, J. A. Lane, E. L. Turner, D. Neal, F. C. Hamdy, R. M. Martin, J. L. Donovan, for the ProtecT study group, Prasad Bollina, Andrew Doble, Alan Doherty, David Gillatt, Vincent Gnanapragasam, Owen Hughes, Roger Kockelbergh, Howard Kynaston, Alan Paul, Edgar Paez, Edward Rowe

**Affiliations:** 1 Health Economics Bristol (HEB), Population Health Sciences, Bristol Medical School, University of Bristol, Bristol, BS8 1NU UK; 2 NIHR Collaboration for Leadership in Applied Health Research and Care West at University Hospitals Bristol, Bristol, BS1 2NT UK; 3 Bristol Randomised Trials Collaboration, University of Bristol, Bristol, BS8 2PS UK; 4 Population Health Sciences, Bristol Medical School, University of Bristol, Bristol, BS8 2PS UK; 5 Nuffield Department of Surgical Sciences, University of Oxford, Oxford, OX3 7DQ UK; 6 National Institute for Health Research (NIHR) Bristol Biomedical Research Centre, University Hospitals Bristol NHS Foundation Trust and the University of Bristol, Bristol, BS8 2PS UK

**Keywords:** Prostate cancer, ProtecT trial, Lifetime cost-effectiveness, Active monitoring, Radiotherapy, Prostatectomy

## Abstract

**Background:**

Optimal management strategies for clinically localised prostate cancer are debated. Using median 10-year data from the largest randomised controlled trial to date (ProtecT), the lifetime cost-effectiveness of three major treatments (radical radiotherapy, radical prostatectomy and active monitoring) was explored according to age and risk subgroups.

**Methods:**

A decision-analytic (Markov) model was developed and informed by clinical input. The economic evaluation adopted a UK NHS perspective and the outcome was cost per Quality-Adjusted Life Year (QALY) gained (reported in UK£), estimated using EQ-5D-3L.

**Results:**

Costs and QALYs extrapolated over the lifetime were mostly similar between the three randomised strategies and their subgroups, but with some important differences. Across all analyses, active monitoring was associated with higher costs, probably associated with higher rates of metastatic disease and changes to radical treatments.

When comparing the value of the strategies (QALY gains and costs) in monetary terms, for both low-risk prostate cancer subgroups, radiotherapy generated the greatest net monetary benefit (£293,446 [95% CI £282,811 to £299,451] by D’Amico and £292,736 [95% CI £284,074 to £297,719] by Grade group 1). However, the sensitivity analysis highlighted uncertainty in the finding when stratified by Grade group, as radiotherapy had 53% probability of cost-effectiveness and prostatectomy had 43%. In intermediate/high risk groups, using D’Amico and Grade group > = 2, prostatectomy generated the greatest net monetary benefit (£275,977 [95% CI £258,630 to £285,474] by D’Amico and £271,933 [95% CI £237,864 to £287,784] by Grade group). This finding was supported by the sensitivity analysis.

Prostatectomy had the greatest net benefit (£290,487 [95% CI £280,781 to £296,281]) for men younger than 65 and radical radiotherapy (£201,311 [95% CI £195,161 to £205,049]) for men older than 65, but sensitivity analysis showed considerable uncertainty in both findings.

**Conclusion:**

Over the lifetime, extrapolating from the ProtecT trial, radical radiotherapy and prostatectomy appeared to be cost-effective for low risk prostate cancer, and radical prostatectomy for intermediate/high risk prostate cancer, but there was uncertainty in some estimates. Longer ProtecT trial follow-up is required to reduce uncertainty in the model.

**Trial registration:**

Current Controlled Trials number, ISRCTN20141297: http://isrctn.org (14/10/2002); ClinicalTrials.gov number, NCT02044172: http://www.clinicaltrials.gov (23/01/2014).

## Background

Prostate cancer is the most common cancer in men in Europe and the second most common cancer in men worldwide [1], placing a considerable burden on healthcare resources globally. Thirteen percent of all cancer deaths in men in the UK are attributable to prostate cancer [1]. However, due to the typically slow natural progression of the disease, many men with prostate cancer die of other causes, which has led to an international debate about the optimal disease management strategy [2].

UK guidelines developed by the National Institute for Health and Care Excellence (NICE) were recently updated to include evidence from the Prostate Testing for Cancer and Treatment (ProtecT) trial [3]. The guidelines recommend treatment stratified according to the D’Amico risk categories [4, 5]. NICE recommend offering a choice of treatment (active surveillance, prostatectomy and radiotherapy) for men with low risk, localised prostate cancer. For men with intermediate risk, localised prostate cancer, NICE recommend that radical treatment (either surgery or radiotherapy) be offered, but active surveillance should be considered for men who do not want radical treatment. For high risk localised prostate cancer, radical treatment should be offered rather than active surveillance. However, evidence from a direct comparison of the long-term cost-effectiveness of radical prostatectomy, radical radiotherapy and active monitoring for men with localised prostate cancer was not available for all men with localised prostate cancer or by risk stratification, and the recommendations were therefore based on limited direct evidence of cost-effectiveness.

ProtecT is the largest randomised controlled trial to date comparing the effectiveness and cost-effectiveness of active monitoring, radical prostatectomy or external beam 3D conformal radiotherapy with neo-adjuvant androgen deprivation for men with clinically localised prostate cancer detected following PSA testing and transrectal ultrasound guided biopsies [3]. The primary analysis was conducted at a median follow-up period of 10 years.

The aim was to determine the lifetime cost-effectiveness of managing localised prostate cancer according to the following sub-groups: i) age: less than 65 years old, 65 years and older; ii) the D’Amico prostate cancer risk stratification groups: low (PSA < 10 ng/ml, Gleason score < =6, T1c/T2a) or intermediate/high risk ((PSA > =10 ng/ml & < 20 ng/ml, Gleason score 3 + 4 = 7 or 4 + 3 = 7, T2b) or (PSA > =20 ng/ml, Gleason score > =8, T2c)) [4] and iii) by Gleason Grade Group (GGG): low: GGG 1; or intermediate/high risk (GGG > =2) [6]

## Methods

To estimate the lifetime cost-effectiveness of management strategies for clinically localised prostate cancer according to risk group, a model-based cost-effectiveness analysis was conducted using median 10-year follow-up data from the ProtecT trial. The outcome was cost per quality-adjusted life year (QALY) gained based on EQ-5D-3L. A UK NHS perspective was adopted.

### ProtecT trial

The ProtecT trial methods and median 10-year outcomes are reported in detail elsewhere [3, 7] At a median of 10-years’ follow up, the ProtecT trial reported no evidence of differences in prostate-cancer specific mortality [8] between the three management strategies (all approximately 1%), but the rate of disease progression (evidence of clinical progression (T3 or T4 or the initiation of long-term androgen deprivation therapy (ADT)) and metastases in the prostatectomy and radiotherapy group was half that of men in the active monitoring group (6% compared with 2–3%) [9]. There was no evidence of differences in generic health status (mental or physical health, anxiety or depression) between the groups, but prostatectomy had greater adverse effects on sexual function and continence, and radiotherapy on sexual and bowel function [10] compared with active monitoring over the 6-year data collection period.

### Cost-effectiveness model

**Fig. 1:**
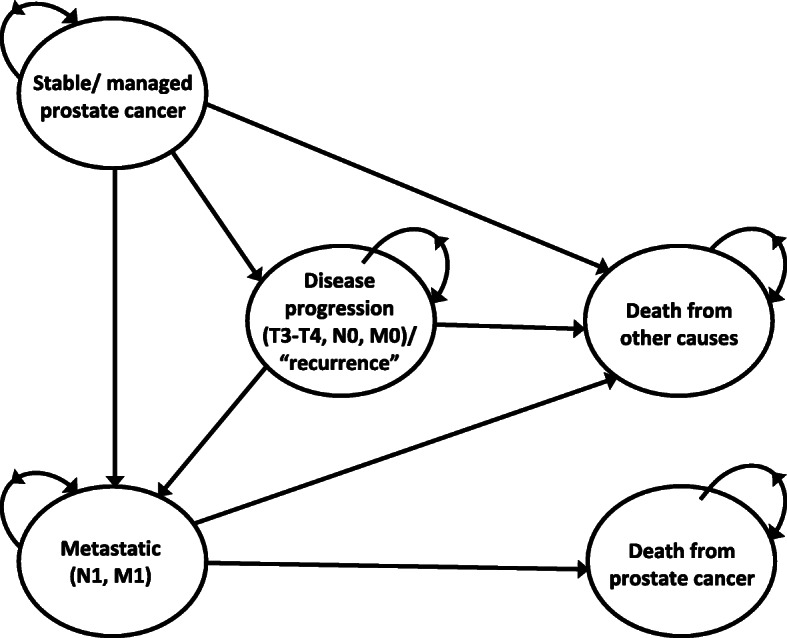
Schematic diagram of the Markov model

We developed a state transition Markov model in Microsoft Excel, programmed in Visual Basic for Applications, to estimate the lifetime cost-effectiveness of alternative treatment strategies for men with clinically localised prostate cancer. Clinical input from the ProtecT investigators on the pathways followed by men in the ProtecT trial informed the model structure (Fig. 1). In the model men could: remain in a stable or managed prostate cancer state where men are receiving one of the randomised strategies; experience local, or distant disease progression (metastases). The disease progression and metastatic states followed the same definitions as in the ProtecT trial: evidence of clinical progression (T3 or T4 disease or the initiation of long-term androgen deprivation therapy (ADT) or presence of metastasis (M1, N1), PSA > 100 μg/ml) [7]. From any state, a man could die of causes other than prostate cancer. Men might remain in the same state or move between health states at annual intervals, reflecting the slow progressive nature of most prostate cancers.

### Parameter estimation

Model inputs for the transition of men between health states were estimated from the ProtecT trial patient-level data (Tables S1, S3, S5 in supplementary material), with starting ages of 50–69 years, and applied to the model cohort with a starting age of 50 years. To estimate death due to other causes, deaths related to prostate cancer (defined as ‘ICD C61: malignant neoplasm of prostate’) were removed from the UK national life tables. As expected, the estimate of death due to other causes increased as the age of men in the model increased.

The following assumptions were made based on clinical expertise within the ProtecT team, and were agreed prior to conducting the analysis:

- The initial and subsequent treatment received by men in the ‘stable’, ‘disease progression’ and ‘metastatic’ states followed what was observed in the trial;
- The same quality of life and resource use observed during trial follow-up for each health state in the model was assumed to continue beyond the first ten years;
- For simplicity, it was only possible for a man to die from prostate cancer from the metastatic state in the model. While it is possible for a man to die from local progression, this is uncommon;
- The same costs were assumed to have occurred in the last year of life across the treatment strategy;
- The disease progression state comprised men found to have tumour stage T3-T4, or those treated with hormone therapy, or where clinicians indicated that the disease had progressed locally.

### Quality of life

Self-reported quality of life data were collected in the ProtecT trial using EQ-5D-3L at baseline (prior to diagnosis), 6 months, and yearly thereafter [9]. Quality of life values were assigned to each health state and the algorithm applied for EQ-5D-3L UK value set [11] (Tables S2, S4, S6 in supplementary material). Quality of life scores were calculated by averaging the score of men in a health state at any given time. For missing scores, the mean of the adjacent years (prior and following) were used. The outcome was reported as QALYs.

### Resource use and costs

Costs were presented from a UK NHS perspective. Data on healthcare resource use were collected from men and a local medical records review in the within-trial analysis [12]. Data on costs and resource use from the trial were collated and analysed according to the randomised treatment arm (Tables S2, S4, S6 in supplementary material). The general healthcare costs for the groups included inpatient and day-case hospital stays, outpatient hospital visits, GP visits, healthcare staff costs and medication costs. Costs and outcomes were discounted at 3.5% according to UK NICE guidelines, as the model time horizon extended beyond one year [13]. All costs were reported in 2014–15 price year in UK (£) sterling. As 2015 was the data lock point.

### Analysis

The cost-effectiveness analysis provided an assessment of differences in costs and outcomes between the three treatment options over a man’s lifetime from the time a diagnosis of prostate cancer was made. As three management strategies were compared, the results are presented as net monetary benefits. The net monetary benefit is the value of the strategy in monetary terms at a specific willingness to pay threshold per unit of benefit gained. An average UK (NICE) willingness-to-pay threshold of £20,000 per QALY gained was used. The strategy with the greatest net monetary benefit ((*Benefit* × **threshold**) − *Cost*) is considered to be the most cost-effective strategy. When comparing the strategies, the evidence that the cost-effectiveness of strategies differs is weak if the confidence intervals overlap.

Two types of parametric proportional hazards models were used to extrapolate annual mean transition estimates beyond the trial. Weibull models, which assume a changing rate of transition over time, had the best fit for stable to local disease progression and stable to metastases transitions. The exponential models, which assume a constant rate of transition, fitted the data best for local disease progression to metastases and metastases to death from prostate cancer. Patient-level trial data were used to fit the models. For the quality of life data, we used the average value across the three management strategies for each state; therefore, the impact on quality of life is captured through the proportions of men entering each state by management strategy.

Analysis was carried out by intention-to-treat. The following analyses were performed:

- Stratification by age to compare cost-effectiveness for men less than 65 years old and 65 years or older (Table S1–2 in supplementary material).
- Stratification by D’Amico risk groups to compare cost-effectiveness for low risk and combined intermediate/high risk groups (Table S3–4 in supplementary material).

- Stratification by Grade groups to compare cost-effectiveness for low risk (GGG 1) and combined intermediate/high risk groups (GGG > = 2) (Table S5–6 in supplementary material). Intermediate and high risk subgroups were combined due to small numbers.

- A probabilistic sensitivity analysis of the above groups to provide a more comprehensive assessment of uncertainty, as all the relevant model inputs are changed simultaneously rather than changing one input at a time. For each model input, a distribution of plausible range of values was assigned. A value was then randomly drawn from the assigned distribution. This process was repeated 10,000 times to obtain a measure of uncertainty around the results, which are presented as cost-effectiveness acceptability curves (CEACs) to illustrate how the willingness-to-pay threshold for an additional QALY affects the probability that a management strategy is considered cost-effective.

## Results

**Fig. 2:**
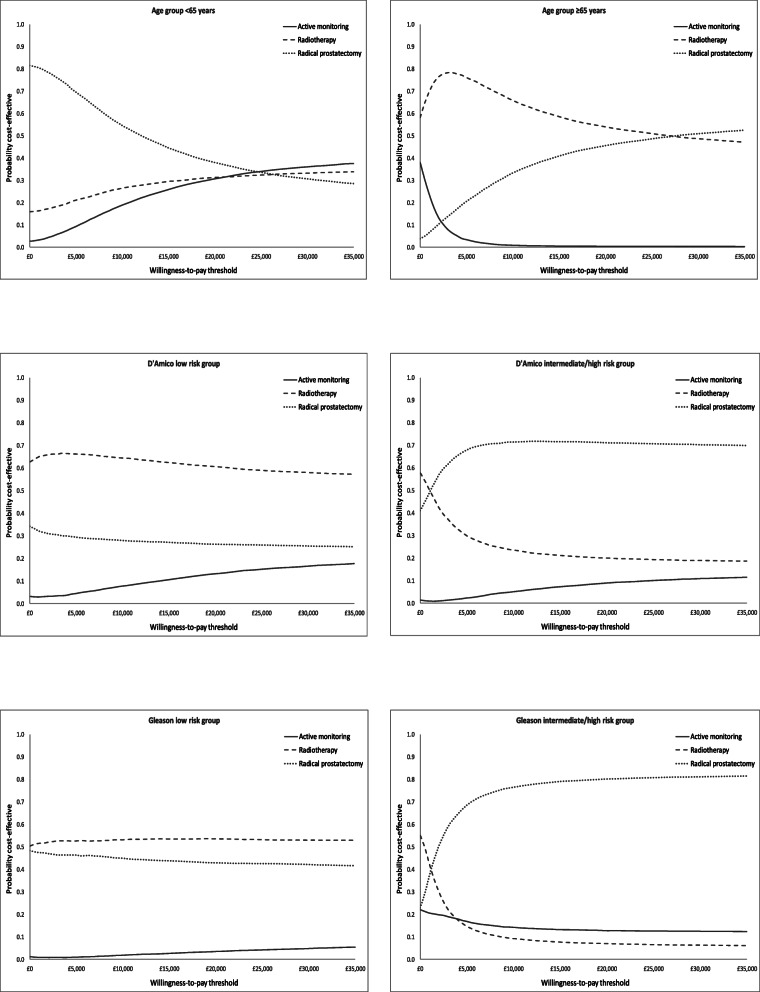
Cost-effectiveness acceptability curves for all analyses

Table 1 presents the cost-effectiveness results for the different risk groups and Fig. 2 presents the uncertainty analysis in the CEAC. Costs and QALYs were similar across treatment groups with some key differences.

**Table 1:** Cost-effectiveness results from age and risk stratification analyses

Strategy	Mean lifetime cost per patient^**1**^	Mean lifetime QALYs per patient^**1**^	NMB^**2**^ at £20,000 per QALY (95% CI)
**Age group < 65 years**			
Active monitoring (AM)^3^	£15,359	15.36	£290,279 (£281,895 to £295,127)
Radical prostatectomy (RP)	£12,939	15.25	**£290,487 (£280,781 to £296,281**)*
Radiotherapy (RT)	£14,746	15.32	£289,754 (£278,620 to £296,202)
**Age group ≥ 65 years**			
Active monitoring (AM)	£9444	10.23	£194,153 (£186,516 to £199,781)
Radical prostatectomy (RP)^4^	£10,283	10.61	£201,052 (£195,294 to £204,579)
Radiotherapy (RT)	£9174	10.57	£201,311 (£195,161 to £205,049)*
**D’Amico low risk group**			
Active monitoring (AM)	£14,027	15.29	£289,965 (£279,855 to £296,013)
Radical prostatectomy (RP)	£12,072	15.25	£290,967 (£279,084 to £297,907)
Radiotherapy (RT)	£11,572	15.34	£293,446 (£282,811 to £299,451)*
**D’Amico intermediate/high risk group**			
Active monitoring (AM)	£18,297	14.30	£265,526 (£247,010 to £278,307)
Radical prostatectomy (RP)	£15,323	14.70	£275,977 (£258,630 to £285,474)*
Radiotherapy (RT)	£15,060	14.35	£268,669 (£246,778 to £282,238)
**Grade group - low risk**			
Active monitoring (AM)	£14,144	15.15	£287,565 (£278,750 to £293,382)
Radical prostatectomy (RP)	£12,055	15.28	£292,198 (£283,797 to £297,258)
Radiotherapy (RT)	£12,041	15.31	£292,736 (£284,074 to £297,719)*
**Grade group - intermediate/high risk**			
Active monitoring (AM)	£17,838	13.90	£256,111 (£226,180 to £276,399)
Radical prostatectomy (RP)	£17,645	14.78	£271,933 (£237,864 to £287,784)*
Radiotherapy (RT)	£16,317	13.54	£248,558 (£207,893 to £274,911)

^1^ Deterministic analysis; ^2^ Probabilistic sensitivity analysis; QALYs (quality adjusted life years); NMB (net monetary benefit); CI (confidence interval) based on the percentile method; ^3^ AM becomes more cost-effective compared with both RP and RT beyond a willingness-to-pay (WTP) threshold of £24,000 for age subgroup < 65 years (Fig. 2); ^4^ RP becomes more cost-effective compared with RT beyond a WTP threshold of £28,000 for age subgroup ≥65 years (Fig. 2); *indicates the most cost-effective strategy

### Age thresholds

Over a lifetime, both QALYs gained and costs were greater in men who were younger than 65 years than men older than 65 years. In the age group analysis, there was considerable uncertainty in the results. In men aged less than 65 years, prostatectomy generated the greatest net monetary benefit (£290,487; [95% CI £280,781, £296,281]), but the net monetary benefit was similar for active monitoring (£290,279; [95% CI £281,895, £295,127]). The probabilistic sensitivity analysis (Fig. 2) showed that from a 0-£25,000 per QALY gained decision-maker threshold, prostatectomy was more likely to be cost-effective with a probability of ~ 40% at £20,000 per QALY gained. However, this probability differed only by 10% compared to the other strategies as the likelihood of active monitoring and radiotherapy being cost-effective was approximately 30% for both strategies at the same decision-maker threshold. For men who were older than 65 years old, radiotherapy generated the greatest net monetary benefit (£201,311; [95% CI £195,161 to £205,049]), but the result was similar to prostatectomy (£201,052; [95% CI £195,294 to £204,579]). The probabilistic sensitivity analysis (Fig. 2) showed that radiotherapy was more likely to be cost-effective up to a willingness to pay threshold of £27,000 per QALY gained, but beyond this point prostatectomy becomes more cost-effective. At a threshold of £20,000 per QALY gained, radiotherapy had a 55% probability of being cost-effective compared to 45% for prostatectomy.

### Low risk localised prostate cancer

Over a lifetime, in men with low risk prostate cancer (according to D’Amico risk stratification), radiotherapy was the least costly strategy and generated greater QALYs overall than both active monitoring (£2455 and 0.08 QALY difference) and prostatectomy (£500 and 0.09 QALY difference). The difference in QALY benefit generated by radiotherapy was approximately equivalent to an additional month in perfect health over a man’s lifetime. Radiotherapy therefore marginally generated the greatest net monetary benefit (£293,446; [95% CI £282,811, £299,451]) in low risk prostate cancer and these results were supported by the CEAC (Fig. 2) that showed that radiotherapy had the greatest likelihood of cost-effectiveness at any decision-maker willingness to pay threshold. At a £20,000 per QALY gained threshold, radiotherapy had a ~ 60% likelihood when compared to prostatectomy (~ 30% likelihood) and active monitoring (~ 10%). When stratified by grade group, in the low risk GGG, radiotherapy was marginally less costly (£14) and was associated with marginally more QALY gains (0.03 QALYs) when compared to prostatectomy. However, the net monetary benefit was similar: £292,736 (95% CI £284,074 to £297,719) and £292,198 (95% CI £283,797 to £297,719) for radiotherapy and prostatectomy, respectively. The probabilistic sensitivity analysis showed the extent of the uncertainty in the result as radiotherapy was only slightly more likely to be cost-effective at all decision-maker thresholds – reaching ~ 53% for radiotherapy and ~ 45% for prostatectomy at a £20,000 per QALY gained threshold (Fig. 2).

### Intermediate/high risk localised prostate cancer

For both risk groups (D’Amico and GGG), the QALYs gained were consistently lower in the intermediate/high risk subgroups when compared to the low risk groups. Costs varied between management strategies, ranging between £11,060–£18,297, but were consistently higher in the intermediate/high risk subgroups when compared to low risk groups.

In the D’Amico intermediate/high risk group, active monitoring was more expensive over a lifetime than the radical strategies, which were similar in cost – prostatectomy cost £15,323 and radiotherapy £15,060. However, active monitoring and radiotherapy achieved similar QALY gains, and prostatectomy achieved 0.4 more QALY gains than active monitoring and 0.35 more QALYs than radiotherapy. Prostatectomy generated the greatest net monetary benefit (£275,977; [95% CI £258,630 to £285,474]) for men with intermediate/high risk prostate cancer. The probabilistic sensitivity analysis showed that prostatectomy had a 70% likelihood of being cost-effective from a willingness to pay threshold of £5000 per QALY gained upward. Whilst radiotherapy and active monitoring only had 20 and 10%, respectively from £15,000 per QALY gained onward. The results for the intermediate/high risk GGG mirrored that of the D’Amico intermediate/high risk group with prostatectomy generating the greatest net monetary benefit (£271,933 [95% CI £237,864 to £287,784]) over a lifetime. The probabilistic sensitivity analysis showed that prostatectomy had an 80% likelihood of being cost-effective for the intermediate/high risk GGG compared to 5% for radiotherapy and 15% for active monitoring from a decision-maker threshold of £10,000 per QALY gained onward (Fig. 2).

## Discussion

### Main findings

Across all analyses, costs and QALYs were similar but some important differences emerged. From the point of diagnosis of clinically localized prostate cancer, extrapolating from the median 10-year ProtecT trial findings over the lifetime of a man, showed that there was considerable uncertainty in the most cost-effective management strategy when men were categorised by age group. For 65-year-old men and younger, all three management strategies could be considered to be cost-effective, and for men older than 65 years, either radiotherapy or prostatectomy could be cost-effective under the current UK willingness to pay threshold of £20–30,000 per QALY gained [13]. In all analyses, active monitoring was associated with higher costs, which were probably related to the combined impacts of advanced disease (20% over an average of 10 years) which requires new treatments, and a rate of change to radical treatments of over 50% at an average of 10 years from diagnosis.

When categorised by D’Amico risk group, radiotherapy appeared most cost-effective for men with low risk prostate cancer, compared with active monitoring or radical prostatectomy and when defined as Grade Group 1 alone, either radiotherapy or prostatectomy could be considered cost-effective. For men with intermediate/high risk prostate cancer, categorised by D’Amico or Grade group > = 2, radical prostatectomy was the most cost-effective strategy across all decision-maker’s willingness to pay thresholds from £5000 per QALY gained.

### Comparison with other studies

This is the first study to compare the lifetime cost-effectiveness of managing clinically localised prostate cancer by risk group with active monitoring, external beam radiotherapy with neoadjuvant androgen deprivation, and radical prostatectomy by extrapolating from prospectively collected trial data. In continental Europe other model-based cost-utility analyses have used cohort studies and extrapolation from an earlier trial (SPCG-4 which compared watchful waiting to prostatectomy for clinically detected prostate cancer [14]) to assess the cost-effectiveness of active surveillance or watchful waiting compared with prostatectomy, radiotherapy versus prostatectomy, different modalities of radiotherapy against one another, or different surgical techniques against one another [15]. A review of these studies concluded that across all of these studies only small differences in QALY gains were detected between strategies and limited evidence supported cost-effectiveness recommendations of prostatectomy instead of watchful waiting, brachytherapy over prostatectomy, and newer treatment approaches above traditional methods [15]. The within trial analysis for the ProtecT trial over a 10-year time period showed that radiotherapy was the most cost-effective strategy for all men and the subgroup analysis at 10 years showed that active monitoring was most cost-effective for low risk men (D’Amico and Grade group) and men younger than 65 years old. At 10 years, men with intermediate/high risk (D’Amico and Grade group) and men older than 65 years old, radiotherapy was the most cost-effective strategy [12]. Our lifetime cost-effectiveness results show that, in the longer term, active monitoring is generally more expensive than radical strategies due to the rate of metastasis in active monitoring and treatment changeover, and beyond the 10-year time period, it does not appear to be a cost-effective strategy for low risk subgroups. Furthermore, our results show that despite prostatectomy being the most expensive strategy in the intermediate/high risk subgroups, over a lifetime, prostatectomy generates greater QALY gains than radiotherapy and is the most cost-effective strategy in this subgroup. Finally, a recent study with a US perspective compared prostatectomy and radiotherapy to active surveillance for all men with prostate cancer using published effectiveness data from the ProtecT trial. The authors concluded that active surveillance was cost-effective up to 6 years post-diagnosis, but radiotherapy and prostatectomy were cost-effective at 10 years. This was due to increased costs and lower QALYs associated with active surveillance relative to radical strategies due to the higher rate of metastasis in active surveillance, recurrent prostate biopsy costs and treatment change. However, the latter finding was sensitive to the risk of metastasis [16]. None of these studies assessed the lifetime cost-effectiveness or any cost-effectiveness, according to different risk subgroups, of the three major treatment modalities in the PSA-era using consistent and robust evidence on effectiveness, costs and quality of life from a single randomised controlled trial.

### Strengths and limitations

The strength of the study is that it is based on data from the largest randomised controlled trial in localised prostate cancer to date. The model structure was based on pathways observed in the trial, supported by clinical advice, and model assumptions were agreed prior to analysis. This is the first study to report the lifetime cost-effectiveness of these strategies for localised prostate cancer, where all data on resource use and outcomes used in the model were collected prospectively alongside a trial and to use EQ-5D-3L to generate QALYs in localised prostate cancer.

However, there are limitations related both to this analysis, due to the cost price year relating to the data lock point of the trial which was now 5 years ago, and aspects of the ProtecT trial design and timeline. A key limitation was the reliance on the median 10-year follow up available in ProtecT, with diagnostic and treatment pathways designed in the late 1990s. Longer trial follow up is ongoing to identify potential greater differences in the rate of development of progression and metastases and in mortality, that may arise later in the disease pathway. Further, risk-stratification was based on TRUS-guided biopsies (standard at the time) rather than the more accurate but only recently introduced mpMRI scans [17] in the prostate cancer diagnostic pathway, upstream of taking prostate biopsies. The mpMRI enhanced diagnosis of intermediate and high-risk prostate cancer, and reduced detection of low risk disease might have changed the treatment course for some men. Treatments have evolved since recruitment to Protect (1999–2009). Robot-assisted laparoscopic radical prostatectomy is now the prevalent surgical technique, IMRT, the use of lowdose-rate brachytherapy is increasing and ‘spacers’ are increasingly used in men who receive radiotherapy, to reduce toxicity of radiation, but there is currently no evidence that they offer better clinical and patient reported outcomes [18–20]. Different protocols of Active Surveillance from the one used in ProtecT might also have led to different results in terms of rate of change to radical treatment as well as outcomes.

Finally, given that treatment side-effect profiles are important and differ between the trial groups [7], the use of EQ-5D-3L is another limitation, as it is unlikely to capture issues with urinary, bowel and sexual function associated with these treatments [9].

## Conclusion

The analysis provides evidence that when men with clinically localised prostate cancer diagnosed by PSA testing were categorised by age, there is considerable uncertainty around which management strategy could be adopted. For men with low risk prostate cancer, of the three conventional strategies investigated, radical radiotherapy was more likely to be most cost-effective, but due to the extent of the uncertainty radical prostatectomy could also be considered, depending on the patient stratification used to categorise low risk prostate cancer. The results suggest that based on lifetime cost-effectiveness results, radical prostatectomy for high or intermediate risk prostate cancer appears superior to radiotherapy and active monitoring using median 10-year data.

The findings provide some support for the NICE recommendation to offer a choice of management strategy to men with low risk prostate cancer. For men with intermediate/high risk cancer, NICE recommends radical treatment and our findings support surgery. While the paper outlines the most robust lifetime cost-effectiveness analyses from the trial, the limitations outlined previously suggest that the use of newer diagnostic and treatment pathways must be taken into account. Further longer-term ProtecT trial-follow up with modelling of the impact of these newer strategies is required to elucidate more fully the trade-off between costs and effects for optimal management of screen-detected localised prostate cancer over a man’s lifetime.

## Supplementary information

**Additional file 1 Supplementary material**. Contains supplementary data tables, labeled S1, S2 , S3 etc , used in the analysis of the different sub groups.

## Data Availability

On request to the ProtecT study, we will provide a patient deidentified set of EQ. 5D-3L scores from Baseline (Biopsy) onwards, mortality information and annual costs at the Outpatient, Inpatient, GP and medication level, for prostate cancer related research as per informed consent for researchers within the EU and approval by the ProtecT PIs.

## Abbreviations

ADT: Androgen deprivation therapy
CEAC: Cost-effectiveness acceptability curve
GGG: Gleason grade group
NHS: National Health Service
NICE: National Institute for Health and Care Excellence
NMB: Net monetary benefit
PSA: Prostate specific antigen
QALYs: Quality-adjusted life years
