# Supplementary material for: Modelling the lifetime cost-effectiveness of radical prostatectomy, radiotherapy and active monitoring for men with clinically localised prostate cancer from median 10-year outcomes in the ProtecT randomised trial
Source: BMC Cancer. 2020 Oct 7;20:971. doi: 10.1186/s12885-020-07276-4 (PMC7542698; doi:10.1186/s12885-020-07276-4)
Supplement: Supplementary file 1 — Additional file 1 Supplementary material. Contains supplementary data tables, labeled S1, S2 , S3 etc , used in the analysis of the different sub groups. [file 12885_2020_7276_MOESM1_ESM.docx]

**Table S1. Mean estimates for transitions (age subgroup analysis)**

| **Transition** | Mean estimate | Probabilistic sensitivity analysis distribution* | Source |
| --- | --- | --- | --- |
| **Age<65** |  |  |  |
| **Stable to disease progression** |  |  |  |
| Weibull yearly hazard (Active monitoring)  Weibull ancillary parameter, γ | 0.006  1.424 | Cholesky (-5.181, 0.378 SE)  (0.354, 0.105 SE) | ProtecT trial |
| HR (Prostatectomy vs Active monitoring) | 0.316 | Cholesky (-1.152, 0.289 SE) | ProtecT trial |
| HR (Radiotherapy vs Active monitoring) | 0.327 | Cholesky (-1.117, 0.289 SE) | ProtecT trial |
| **Disease progression to metastatic** |  |  |  |
| Exponential yearly hazard (Active monitoring) | 0.012 | Cholesky (-4.383, 0.577 SE) | ProtecT trial |
| HR (Prostatectomy vs Active monitoring) | 2.351 | Cholesky (0.855, 0.816 SE) | ProtecT trial |
| HR (Radiotherapy vs Active monitoring) | 4.929 | Cholesky (1.595, 0.765 SE) | ProtecT trial |
| **Stable to metastatic** |  |  |  |
| Weibull yearly hazard (Active monitoring)  Weibull ancillary parameter, γ | 0.001  1.461 | Cholesky ( -7.005, 0.803 SE)  (0.379, 0.212SE) | ProtecT trial |
| HR (Prostatectomy vs Active monitoring) | 0.316 | Cholesky (-0.826, 0.289 SE) | ProtecT trial |
| HR (Radiotherapy vs Active monitoring) | 0.795 | Cholesky (-0.229, 0.504 SE) | ProtecT trial |
| **Metastatic to prostate cancer death** |  |  |  |
| Exponential yearly hazard (Active monitoring) | 0.016 | Cholesky (-4.125, 1.155 SE) | ProtecT trial |
| HR (Prostatectomy vs Active monitoring) | 7.446 | Cholesky (2.008, 0.802 SE) | ProtecT trial |
| HR (Radiotherapy vs Active monitoring) | 1.651 | Cholesky (0.501, 1.414 SE | ProtecT trial |
| **Age≥65** |  |  |  |
| **Stable to disease progression** |  |  |  |
| Weibull yearly hazard (Active monitoring)  Weibull ancillary parameter, γ | 0.008  1.454 | Cholesky (-4.796, 0.383 SE)  (0.552, 0.374 SE) | ProtecT trial |
| HR (Prostatectomy vs Active monitoring) | 0.403 | Cholesky (-0.909, 0.290 SE) | ProtecT trial |
| HR (Radiotherapy vs Active monitoring) | 0.453 | Cholesky (-0.791, 0.274 SE) | ProtecT trial |
| **Disease progression to metastatic** |  |  |  |
| Exponential yearly hazard (Active monitoring) | 0.064 | Cholesky (-2.753, 0.333 SE) | ProtecT trial |
| HR (Prostatectomy vs Active monitoring) | 0.309 | Cholesky (-1.175, 0.782 SE) | ProtecT trial |
| HR (Radiotherapy vs Active monitoring) | 0.379 | Cholesky (-0.971, 0.782 SE) | ProtecT trial |
| **Stable to metastatic** |  |  |  |
| Weibull yearly hazard (Active monitoring)  Weibull ancillary parameter, γ | 0.002  1.487 | Cholesky -6.258, 0.818 SE)  (0.397, 0.222 SE) | ProtecT trial |
| HR (Prostatectomy vs Active monitoring) | 0.358 | Cholesky (-1.028, 0.584 SE) | ProtecT trial |
| HR (Radiotherapy vs Active monitoring) | 0.257 | Cholesky (-1.358, 0.651 SE) | ProtecT trial |
| **Metastatic to prostate cancer death** |  |  |  |
| Exponential yearly hazard (Active monitoring) | 0.112 | Cholesky (-2.190, 0.378 SE) | ProtecT trial |
| HR (Prostatectomy vs Active monitoring) | 0.920 | Cholesky (-0.084, 0.802 SE) | ProtecT trial |
| HR (Radiotherapy vs Active monitoring) | 1.709 | Cholesky (0.536, 0.690 SE | ProtecT trial |

^*^The Cholesky decomposition method is used (Briggs et al,2006); ^2^ All Cholesky parameters are on log scale; HR (hazard ratio); SE (standard error); ONS (Office for National Statistics)

**Table S2 Cost and outcome values by age subgroup**

|  | **Age<65** | | **Age≥65** | |
| --- | --- | --- | --- | --- |
| **Health State** | **EQ-5D-3L Mean (SE)*** | **Probabilistic Distribution (EQ-5D-3L)** | **EQ-5D-3L Mean (SE)*** | **Probabilistic Distribution (EQ-5D-3L)** |
| Stable | 0.876 (0.002) | Beta (22459, 3173) | 0.865 (0.003) | Beta (15044, 2342) |
| Disease progression | 0.861 (0.012) | Beta (750, 122) | 0.854 (0.010) | Beta (1058, 181) |
| Metastatic | 0.803 (0.018) | Beta (387, 95) | 0.878 (0.015) | Beta (413, 57) |
| Last year of life | 0.750 (0.090) | Beta (17, 6) | 0.632 (0.131) | Beta (8,5) |
| Death from other causes | 0 | Fixed | 0 | Fixed |
| Death from prostate cancer | 0 | Fixed | 0 | Fixed |
|  | **Mean cost estimate £(SE)*** | **Probabilistic Distribution** | **Mean cost estimate £(SE)*** | **Probabilistic Distribution** |
| **Active monitoring** |  |  |  |  |
| Management strategy | 1,302 (75) | *Gamma (301, 4)* | 883 (74) | *Gamma (144, 6)* |
| Stable | 546 (29) | *Gamma (353, 2)* | 523 (38) | *Gamma (189, 3)* |
| Disease progression | 1,660 (173) | *Gamma (92, 18)* | 1,481 (213) | *Gamma (48, 31)* |
| Metastatic | 1,392 (273) | *Gamma (26, 53)* | 1,062 (215) | *Gamma (24, 44)* |
| Last year of life | 4,141 (1623) | *Gamma (7, 636)* | 4,727 (1218) | *Gamma (15, 314)* |
| Death from other causes | 0 | Fixed | 0 | Fixed |
| Death from prostate cancer | 0 | Fixed | 0 | Fixed |
| **Prostatectomy** |  |  |  |  |
| Management strategy | 4,891 (277) | *Gamma (312, 16)* | 4,844 (405) | *Gamma (143, 34)* |
| Stable | 348 (18) | *Gamma (381, 1)* | 336 (24) | *Gamma (200, 2)* |
| Disease progression | 1,036 (164) | *Gamma (40, 26)* | 1,076 (175) | *Gamma (38, 29)* |
| Metastatic | 3,830 (1221) | *Gamma (10, 389)* | 2,242 (754) | *Gamma (9, 254)* |
| Last year of life | 4,141 (1623) | *Gamma (7, 636)* | 4,727 (1218) | *Gamma (15, 314)* |
| Death from other causes | 0 | Fixed | £0 | Fixed |
| Death from prostate cancer | 0 | Fixed | £0 | Fixed |
| **Radiotherapy** |  |  |  |  |
| Management strategy | 4,855 (283) | *Gamma (295, 16)* | 4,503 (372) | *Gamma (147, 31)* |
| Stable | 301 (16) | *Gamma (368, 1)* | 265 (18) | *Gamma (210, 1)* |
| Disease progression | 1,402 (275) | *Gamma (26, 54)* | 1,196 (212) | *Gamma (32, 37)* |
| Metastatic | 4,222 (1076) | *Gamma (15, 274)* | 1,982 (797) | *Gamma (6, 320)* |
| Last year of life | 4,141 (1623 | *Gamma (7, 636)* | 4,727 (1218) | *Gamma (15, 314)* |
| Death from other causes | 0 | Fixed | 0 | Fixed |
| Death from prostate cancer | 0 | Fixed | 0 | Fixed |

*The analysis was also run where the costs and quality of life scores were the same for both risk groups and only the transition estimates differed by subgroup. This change had minimal impact on the results.

Average costs per health state per arm as observed in the ProtecT trial. For detailed costing see within trial analysis cost-effectiveness analysis (Noble et al, submitted). The same cost was applied to last year of life across the arms due to the small numbers in each arm and because these are not expected to differ by initial treatment.

**Table S3. Mean estimates for transitions (D’Amico subgroup analysis)**

| **Transition** | Mean estimate | Probabilistic sensitivity analysis distribution* | Source |
| --- | --- | --- | --- |
| **Low risk** |  |  |  |
| **Stable to disease progression** |  |  |  |
| Weibull yearly hazard (Active monitoring)  Weibull ancillary parameter, γ | 0.001  1.805 | Cholesky (-6.547, 0.578 SE)  (0.591, 0.129 SE) | ProtecT trial |
| HR (Prostatectomy vs Active monitoring) | 0.397 | Cholesky (-0.923, 0.343 SE) | ProtecT trial |
| HR (Radiotherapy vs Active monitoring) | 0.319 | Cholesky (-1.142, 0.367 SE) | ProtecT trial |
| **Disease progression to metastatic** |  |  |  |
| Exponential yearly hazard (Active monitoring) | 0.009 | Cholesky (-4.730, 1.000 SE) | ProtecT trial |
| HR (Prostatectomy vs Active monitoring) | 5.389 | Cholesky (1.684, 1.225 SE) | ProtecT trial |
| HR (Radiotherapy Active vs monitoring) | 6.576 | Cholesky (1.883, 1.225 SE) | ProtecT trial |
| **Stable to metastatic** |  |  |  |
| Weibull yearly hazard (Active monitoring)  Weibull ancillary parameter, γ | 0.001  1.805 | Cholesky (-6.846, 0.578 SE)  (0.268, 0.129) | ProtecT trial |
| HR (Prostatectomy vs Active monitoring) | 0.420 | Cholesky (-0.868, 0.690 SE) | ProtecT trial |
| HR (Radiotherapy vs Active monitoring) | 0.681 | Cholesky (-0.384, 0.586 SE) | ProtecT trial |
| **Metastatic to prostate cancer death** |  |  |  |
| Exponential yearly hazard (Active monitoring) | 0.052 | Cholesky (-2.960, 0.707 SE) | ProtecT trial |
| HR (Prostatectomy vs Active monitoring) | 2.954 | Cholesky (1.083, 0.913 SE) | ProtecT trial |
| HR (Radiotherapy vs Active monitoring) | 1.148 | Cholesky (0.138, 1.000 SE | ProtecT trial |
| **Intermediate/high risk** |  |  |  |
| **Stable to disease progression** |  |  |  |
| Weibull yearly hazard (Active monitoring)  Weibull ancillary parameter, γ | 0.013  1.970 | Cholesky (-4.312, 0.359 SE)  (0.315, 0.106 SE) | ProtecT trial |
| HR (Prostatectomy vs Active monitoring) | 0.389 | Cholesky (-0.944, 0.289 SE) | ProtecT trial |
| HR (Radiotherapy vs Active monitoring) | 0.411 | Cholesky (-0.890, 0.283 SE) | ProtecT trial |
| **Disease progression to metastatic** |  |  |  |
| Exponential yearly hazard (Active monitoring) | 0.039 | Cholesky (-3.245, 0.378 SE) | ProtecT trial |
| HR (Prostatectomy vs Active monitoring) | 0.360 | Cholesky (1.021, 0.802 SE) | ProtecT trial |
| HR (Radiotherapy vs Active monitoring) | 0.734 | Cholesky (-0.310, 0.802 SE) | ProtecT trial |
| **Stable to metastatic** |  |  |  |
| Weibull yearly hazard (Active monitoring)  Weibull ancillary parameter, γ | 0.002  1.684 | Cholesky (-6.493, 0.879 SE)  (0.521, 0.212 SE) | ProtecT trial |
| HR (Prostatectomy vs Active monitoring) | 0.360 | Cholesky (-1.021, 0.584 SE) | ProtecT trial |
| HR (Radiotherapy vs Active monitoring) | 0.370 | Cholesky (-0.994, 0.584 SE) | ProtecT trial |
| **Metastatic to prostate cancer death** |  |  |  |
| Exponential yearly hazard (Active monitoring) | 0.044 | Cholesky (-3.113, 0.577 SE) | ProtecT trial |
| HR (Prostatectomy vs Active monitoring) | 2.034 | Cholesky (0.710, 0.913 SE) | ProtecT trial |
| HR (Radiotherapy vs Active monitoring) | 4.172 | Cholesky (1.428, 0.913 SE | ProtecT trial |

* The Cholesky decomposition method is used (Briggs et al 2006); ^2^ All Cholesky parameters are on log scale; HR (hazard ratio); SE (standard error); ONS (Office for National Statistics)

**Table S4.** **Cost and outcome values by D’Amico risk subgroup**

|  | **Low risk** | | **Intermediate/high risk** | |
| --- | --- | --- | --- | --- |
| **Health State** | **EQ-5D-3L Mean (SE)*** | **Probabilistic Distribution (EQ-5D-3L)** | **EQ-5D-3L Mean (SE)*** | **Probabilistic Distribution (EQ-5D-3L)** |
| Stable | 0.879 (0.002) | Beta (24756, 3406) | 0.854 (0.003) | Beta (9783, 1678) |
| Disease progression | 0.843 (0.016) | Beta (420, 78) | 0.844 (0.012) | Beta (786, 145) |
| Metastatic | 0.831 (0.018) | Beta (359, 73) | 0.852 (0.019) | Beta (286, 50) |
| Last year of life | 0.660 (0.155) | Beta (3, 3) | 0.666 (0.161) | Beta (5,3) |
| Death from other causes | 0 | Fixed | 0 | Fixed |
| Death from prostate cancer | 0 | Fixed | 0 | Fixed |
|  | **Mean cost estimate £(SE)*** | **Probabilistic Distribution** | **Mean cost estimate £(SE)*** | **Probabilistic Distribution** |
| **Active monitoring** |  |  |  |  |
| Management strategy | 928 (60) | *Gamma (236, 4)* | 1467 (116) | *Gamma (160, 9)* |
| Stable | 464 (24) | *Gamma (386, 1)* | 729 (57) | *Gamma (164, 4)* |
| Disease progression | 2,117 (306) | *Gamma (48, 44)* | 1,459 (182) | *Gamma (64, 23)* |
| Metastatic | 1,093 (294) | *Gamma (14, 79)* | 1,327 (258) | *Gamma (26, 50)* |
| Last year of life | 4,464 (1502) | *Gamma (9, 505)* | 3,365 (1148) | *Gamma (9, 392)* |
| Death from other causes | 0 | Fixed | 0 | Fixed |
| Death from prostate cancer | 0 | Fixed | 0 | Fixed |
| **Prostatectomy** |  |  |  |  |
| Management strategy | 4,654 (296) | *Gamma (248, 19)* | 5,335 (424) | *Gamma (158, 34)* |
| Stable | 303 (15) | *Gamma (405, 1)* | 398 (30) | *Gamma (177, 2)* |
| Disease progression | 1,242 (278) | *Gamma (20, 62)* | 990 (135) | *Gamma (54, 18)* |
| Metastatic | 4,063 (1590) | *Gamma (7, 622)* | 2,618 (869) | *Gamma (9, 288)* |
| Last year of life | 4,464 (1502) | *Gamma (9, 505)* | 3,365 (1148) | *Gamma (9, 392)* |
| Death from other causes | 0 | Fixed | £0 | Fixed |
| Death from prostate cancer | 0 | Fixed | £0 | Fixed |
| **Radiotherapy** |  |  |  |  |
| Management strategy | 4,653 (295) | *Gamma (248, 19)* | 4,902 (396) | *Gamma (153, 32)* |
| Stable | 264 (13) | *Gamma (416, 1)* | 331 (25) | *Gamma (179, 2)* |
| Disease progression | 1159 (289) | *Gamma (16, 72)* | 1,696 (323) | *Gamma (27, 62)* |
| Metastatic | 3,222 (917) | *Gamma (12, 261)* | 2,203 (1212) | *Gamma (3, 667)* |
| Last year of life | 4,464 (1502) | *Gamma (9, 505)* | 3,365 (1148) | *Gamma (9, 392)* |
| Death from other causes | 0 | Fixed | 0 | Fixed |
| Death from prostate cancer | 0 | Fixed | 0 | Fixed |

*The analysis was also run where the costs and quality of life scores were the same for both risk groups and only the transition estimates differed by subgroup. This change had minimal impact on the results.

Average costs per health state per arm as observed in the ProtecT trial. For detailed costing see within trial analysis cost-effectiveness analysis (Noble et al, submitted). The same cost was applied to last year of life across the arms due to the small numbers in each arm and because these are not expected to differ by initial treatment.

**Table S5. Mean estimates for transitions (Grade group subgroup)**

| **Transition** | Mean estimate | Probabilistic sensitivity analysis distribution* | Source |
| --- | --- | --- | --- |
| **Low risk** |  |  |  |
| **Stable to disease progression** |  |  |  |
| Weibull yearly hazard (Active monitoring)  Weibull ancillary parameter, γ | 0.003  1.613 | Cholesky (-5.775, 0.404 SE)  (0.478, 0.100 SE) | ProtecT trial |
| HR (Prostatectomy vs Active monitoring) | 0.300 | Cholesky (-1.204, 0.287 SE) | ProtecT trial |
| HR (Radiotherapy vs Active monitoring) | 0.358 | Cholesky (-1.027, 0.269 SE) | ProtecT trial |
|  |  |  |  |
| **Disease progression to metastatic** |  |  |  |
| Exponential yearly hazard (Active monitoring) | 0.019 | Cholesky (-3.966, 0.500 SE) | ProtecT trial |
| HR (Prostatectomy vs Active monitoring) | 1.366 | Cholesky (0.312, 0.866 SE) | ProtecT trial |
| HR (Radiotherapy vs Active monitoring) | 1.787 | Cholesky (0.580, 0.0.764 SE) | ProtecT trial |
|  |  |  |  |
| **Stable to metastatic** |  |  |  |
| Weibull yearly hazard (Active monitoring)  Weibull ancillary parameter, γ | 0.001  1.338 | Cholesky (-6.823, 0.743 SE)  (0.291, 0.213 SE) | ProtecT trial |
| HR (Prostatectomy vs Active monitoring) | 0.399 | Cholesky (-0.919, 0.592 SE) | ProtecT trial |
| HR (Radiotherapy vs Active monitoring) | 0.598 | Cholesky (-0.515, 0.516 SE) | ProtecT trial |
|  |  |  |  |
| **Metastatic to prostate cancer death** |  |  |  |
| Exponential yearly hazard (Active monitoring) | 0.052 | Cholesky (-2.959, 0.577 SE) | ProtecT trial |
| HR (Prostatectomy vs Active monitoring) | 2.660 | Cholesky (0.978, 0.816 SE) | ProtecT trial |
| HR (Radiotherapy vs Active monitoring) | 1.006 | Cholesky (0.006, 0.913 SE | ProtecT trial |
| **Intermediate/high risk** |  |  |  |
| **Stable to disease progression** |  |  |  |
| Weibull yearly hazard (Active monitoring)  Weibull ancillary parameter, γ | 0.008  1.643 | Cholesky (-4.859, 0.560 SE)  (0.496, 0.143 SE) | ProtecT trial |
| HR (Prostatectomy vs Active monitoring) | 0.321 | Cholesky (-1.137, 0.393 SE) | ProtecT trial |
| HR (Radiotherapy vs Active monitoring) | 0.387 | Cholesky (-0.950, 0.393 SE) | ProtecT trial |
|  |  |  |  |
| **Disease progression to metastatic** |  |  |  |
| Exponential yearly hazard (Active monitoring) | 0.052 | Cholesky (-2.948, 0.447 SE) | ProtecT trial |
| HR (Prostatectomy vs Active monitoring) | 0.560 | Cholesky (-0.580, 0.837 SE) | ProtecT trial |
| HR (Radiotherapy vs Active monitoring) | 1.350 | Cholesky (0.300, 0.837 SE) | ProtecT trial |
|  |  |  |  |
| **Stable to metastatic** |  |  |  |
| Weibull yearly hazard (Active monitoring)  Weibull ancillary parameter, γ | 0.001  2.078 | Cholesky (-7.20, 1.307 SE)  (0.731, 0.262 SE) | ProtecT trial |
| HR (Prostatectomy vs Active monitoring) | 0.130 | Cholesky (-2.038, 1.069 SE) | ProtecT trial |
| HR (Radiotherapy vs Active monitoring) | 0.627 | Cholesky (-0.467, 0.627 SE) | ProtecT trial |
|  |  |  |  |
| **Metastatic to prostate cancer death** |  |  |  |
| Exponential yearly hazard (Active monitoring) | 0.065 | Cholesky (-2.733, 0.577 SE) | ProtecT trial |
| HR (Prostatectomy vs Active monitoring) | 2.002 | Cholesky (0.694, 1.155 SE) | ProtecT trial |
| HR (Radiotherapy vs Active monitoring) | 2.854 | Cholesky (1.049, 0.913 SE) | ProtecT trial |

* The Cholesky decomposition method is used (Briggs et al 2006); ^2^ All Cholesky parameters are on log scale; HR (hazard ratio); SE (standard error); ONS (Office for National Statistics)

**Table S6.** **Cost and outcome values by Grade group subgroup**

|  | **Low risk** | | **Intermediate/high risk** | |
| --- | --- | --- | --- | --- |
| **Health State** | **EQ-5D-3L Mean (SE)*** | **Probabilistic Distribution (EQ-5D-3L)** | **EQ-5D-3L Mean (SE)*** | **Probabilistic Distribution (EQ-5D-3L)** |
| Stable | 0.875 (0.002) | Beta (29905, 4281) | 0.863 (0.004) | Beta (7417, 1181) |
| Disease progression | 0.858 (0.011) | Beta (809, 133) | 0.855 (0.011) | Beta (880, 147) |
| Metastatic | 0.815 (0.017) | Beta (403, 92) | 0.856 (0.017) | Beta (357, 57) |
| Last year of life | 0.652 (0.120) | Beta (10, 5) | 0.890 (0.138) | Beta (7,3) |
| Death from other causes | 0 | Fixed | 0 | Fixed |
| Death from prostate cancer | 0 | Fixed | 0 | Fixed |
|  | **Mean cost estimate £(SE)*** | **Probabilistic Distribution** | **Mean cost estimate £(SE)*** | **Probabilistic Distribution** |
| **Active monitoring** |  |  |  |  |
| Management strategy | 1,013 (57) | *Gamma (321, 3)* | 1356 (147) | *Gamma (85, 16)* |
| Stable | 507 (24) | *Gamma (437, 1)* | 562 (56) | *Gamma (102, 6)* |
| Disease progression | 1,743 (195) | *Gamma (80, 22)* | 1,722 (272) | *Gamma (40, 43)* |
| Metastatic | 1,153 (255) | *Gamma (21, 56)* | 1,401 (277) | *Gamma (26, 55)* |
| Last year of life | 5,207 (1471) | *Gamma (13, 415)* | 5,174 (1760) | *Gamma (9, 599)* |
| Death from other causes | 0 | Fixed | 0 | Fixed |
| Death from prostate cancer | 0 | Fixed | 0 | Fixed |
| **Prostatectomy** |  |  |  |  |
| Management strategy | 4,727 (262) | *Gamma (325, 15)* | 5,391 (550) | *Gamma (96, 56)* |
| Stable | 317 (15) | *Gamma (458, 1)* | 460 (41) | *Gamma (126, 4)* |
| Disease progression | 1,147 (203) | *Gamma (32, 36)* | 982 (189) | *Gamma (27, 36)* |
| Metastatic | 3,723 (1388) | *Gamma (7, 518)* | 5,428 (2396) | *Gamma (5, 1058)* |
| Last year of life | 5,207 (1471) | *Gamma (13, 415)* | 5,174 (1760) | *Gamma (9, 392)* |
| Death from other causes | 0 | Fixed | £0 | Fixed |
| Death from prostate cancer | 0 | Fixed | £0 | Fixed |
| **Radiotherapy** |  |  |  |  |
| Management strategy | 4,642 (259) | *Gamma (248, 19)* | 5,037 (587) | *Gamma (74, 68)* |
| Stable | 277 (13) | *Gamma (416, 1)* | 308 (30) | *Gamma (103, 3)* |
| Disease progression | 1053 (173) | *Gamma (16, 72)* | 1,999 (578) | *Gamma (12, 167)* |
| Metastatic | 3,353 (907) | *Gamma (12, 261)* | 2,203 (972) | *Gamma (3, 667)* |
| Last year of life | 5,207 (1471) | *Gamma (13, 415)* | 5,174 (1760) | *Gamma (5, 429)* |
| Death from other causes | 0 | Fixed | 0 | Fixed |
| Death from prostate cancer | 0 | Fixed | 0 | Fixed |

*The analysis was also run where the costs and quality of life scores were the same for both risk groups and only the transition estimates differed by subgroup. This change had minimal impact on the results.

Average costs per health state per arm as observed in the ProtecT trial. For detailed costing see within trial analysis cost-effectiveness analysis (Noble et al, submitted). The same cost was applied to last year of life across the arms due to the small numbers in each arm and because these are not expected to differ by initial treatment.

**Reference**

Briggs A, Claxton K, Sculpher M. Decision Modelling for Health Economic Evaluation. Oxford:Oxford University Press; 2006.
